# Evaluating the Effectiveness of Noninvasive Ventilation in Patients With Acute Respiratory Failure Due to Pneumonia

**DOI:** 10.7759/cureus.83423

**Published:** 2025-05-03

**Authors:** Zarmast Khan, Nosheen Zabih Noorbaksh, Sajid Hussain Sherazi, Abdul Manan, Ayesha Iqbal, Amna Iqbal Butt, Rehan Aslam, Marriam Khan

**Affiliations:** 1 Pediatric Medicine, Niazi Medical & Dental College, Sargodha, PAK; 2 Obstetrics and Gynecology, Ayub Medical College, Abbottabad, PAK; 3 Internal Medicine, Alkhidmat Raazi Hospital, Rawalpindi, PAK; 4 Pharmacology, Sharif Medical and Dental College, Lahore, PAK; 5 Community Medicine, Sharif Medical and Dental College, Lahore, PAK; 6 Internal Medicine, Islamic International Medical College, Rawalpindi, PAK; 7 Medicine, Hamdard University, Karachi, PAK

**Keywords:** acute respiratory failure, icu admission, mortality, noninvasive ventilation, oxygenation, pneumonia

## Abstract

Respiratory failure is a complication of pneumonia known to contribute substantially to worldwide morbidity and mortality, given that, in most cases, it necessitates ventilatory support to maximize oxygenation and prevent deterioration of the patient’s clinical status. Noninvasive ventilation (NIV) has emerged as a possible alternative to invasive mechanical ventilation (IMV) with noteworthy advantages, including reduced ICU admissions, lower intubation rates, and fewer ventilator-associated complications. This retrospective study aims to analyze the efficacy of NIV in promoting clinical outcomes in pneumonia patients suffering from acute respiratory failure (ARF). A total of 840 patients were analyzed. The mean age was 52.62 years (SD = 21.08), with equal distribution concerning gender. There was a mean distribution of 2.53 comorbidities (SD = 1.66), with an average duration of symptoms of 15.21 days (SD = 8.13) before admission. Oxygenation parameters increased significantly following NIV intervention, with a mean SpO₂ post-treatment of 90.31% (SD = 5.68) and a mean PaO₂ of 74.64 mmHg (SD = 14.69). Clinical outcomes showed that NIV achieved an average decrease in ICU admission of 49.3% and an average decrease in intubation rates of 50.7%. The mortality rate remained very high at 49.7%, while the readmission rate stood at 50.0%, suggesting a continuing clinical hazard even after the patient recovered for some time. Quality-of-life scores averaged 5.74 on a 10-point scale, indicating moderate improvement after treatment. There was also evidence that more than half of the patients required rehabilitation support after discharge from the hospital. This highlights the paramount need for structured post-acute care. NIV has drawbacks, as it was not efficacious for all patients, and patient selection criteria, timing of intervention, and severity of illness heavily influenced the outcomes of treatment. There is a definite promise for NIV to be used as the first-line intervention for ARF associated with pneumonia, as it improves oxygenation, reduces the necessity for IMV, and assists recovery post-treatment. Further studies are required to standardize ventilatory regimens, triage patients, and investigate long-term outcomes. Therefore, because of its advantages in clinical care, NIV should be considered a critical management modality for treating respiratory failure due to pneumonia.

## Introduction

Acute respiratory failure (ARF) is a potentially fatal illness where the respiratory system is unable to eliminate carbon dioxide from the blood or provide enough oxygen [1]. Any number of causes may be behind it, such as pneumonia, chronic obstructive pulmonary disease (COPD), and acute respiratory distress syndrome (ARDS) [2]. An infection known as pneumonia causes inflammation in one or both of the lungs' air sacs. This pneumonia is still the most common cause of significant morbidity and mortality around the globe. Pneumonia-related ARF is, therefore, a big-time and urgent public health issue that necessitates timely and effective intervention to bring about better outcomes for patients and a reduction of complications [3]. For many years, the primary intervention for respiratory failure treatment has included invasive mechanical ventilation (IMV)-based treatment methods.

IMV is associated with different types of complications, including but not limited to ventilator-associated pneumonia (VAP), barotrauma, and prolonged ICU stays [4]. In recent years, noninvasive ventilation (NIV) has become an alternative for ventilatory management, but without the complications caused by endotracheal intubation [5]. NIV, therefore, refers to positive pressure ventilation through a face mask or a nasal interface, thereby minimizing the invasive delivery method while improving gas exchange and respiratory mechanics [6]. The pathophysiology of ARF brought about by pneumonia is inflammation and infection of the lung parenchyma, leading to impairment of alveolar gas exchange, hypoxemia, and respiratory distress [7]. The development of the inflammatory response is associated with the accumulation of fluid and cellular debris in the alveoli and leads to reduced lung compliance and increased work of breathing [8]. It results progressively in respiratory failure and the need for ventilatory support. Old age, immunosuppression, chronic lung diseases, and several associated comorbid conditions like diabetes mellitus and cardiovascular disease risk factors were found to be likely to be pneumonia-induced ARF [9]. In interpreting the outcomes of patients in this situation, the severity of pneumonia and the degree of respiratory compromise will be key in evaluating the need for ventilation support. IMV was considered to be the gold standard treatment of very severe ARF. Still, NIV has since become equally well-known as it offers adequate respiratory support with reduced complications arising from invasive ventilation [10].

The current puzzle is facing optimal management of ARF from pneumonia despite the continued advancement of respiratory support technologies [11]. The decision to go for NIV instead of IMV is complicated and requires continuous evaluation of patient-specific parameters, disease severity, and treatment response. Indeed, many research studies have brought forward benefits accruing from NIV among COPD and cardiogenic pulmonary edema patients. However, its use among pneumonia patients with ARF remains an ongoing debate [12]. It is necessary to study the NIV’s efficacy regarding clinical improvements, mortality decrease, and prevention of intubation necessity for patients with ARF secondary to pneumonia. Evidence about NIV efficiency in this population may inform clinical decision-making, optimize treatment strategies, and ultimately lower the healthcare burden of lengthy ICU stays or ventilator-related complications [13].

Studies have shown reduced intubation rates and better survival rates with the use of NIV among patients diagnosed with COPD and cardiogenic pulmonary edema [14]. However, its efficacy during pneumonia resulting in ARF is still debatable. Analysis showed that NIV can provide short-term benefits in some patients with pneumonia-related ARF; the difference in overall impact on mortality and intubation rates is still unclear [15]. NIV enhanced oxygenation and decreased intubation rates among pneumonia patients. High-flow nasal oxygen therapy (HFNOT) was more effective than NIV in some hypoxemic respiratory failure cases. Such mixed evidence demanded further inquiry into the possible involvement of NIV in pneumonia-related ARF. Another relevant area for exploration has involved identifying patient subgroups who might benefit most from NIV, determining predictors of success, and studying optimal ventilation settings.

This study primarily aims to determine the impact of NIV in ARF due to pneumonia. Its specific objectives include examining the effect of NIV on intubation rate and mortality in patients with pneumonia-related ARF, exploring the physiological impacts of NIV on gas exchange and respiratory mechanics and work of breathing, detecting patient characteristics and clinical factors predicting positive outcomes of NIV, comparing the effect of NIV with other forms of respiratory support such as HFNOT and standard oxygen therapy, and evaluating the complication rates associated with NIV for pneumonia-induced ARF. The outcomes of this study will likely provide information regarding the application of NIV in the management of ARF due to pneumonia, thereby complementing the evidence-based clinical practice guidelines for patient-centered care.

## Materials and methods

Study design

The retrospective study was piloted in three tertiary care centers across Punjab, Pakistan, between January 2020 and December 2023. To identify patients, electronic health records were analyzed using the diagnostic codes pertaining to pneumonia and ARF. Included were adult patients (≥18 years) who had a diagnosis of ARF as a consequence of pneumonia and required either noninvasive or invasive ventilation support. Criteria for inclusion comprised clinical diagnosis, arterial blood gas (ABG)-confirmed hypoxemia, radiological findings, and the need for ventilatory support.

Data collection

The dataset is developed to consider every possible patient characteristic to give a performative analysis of treatment outcome factors. Thus, demographic data, including age, sex, BMI, and smoking status, were recorded along with clinical characteristics, including comorbidities, symptom severity, and duration. Diagnostic data are made up of methods of diagnosis, primary and secondary diagnoses, imaging findings from chest X-rays and CT scans, and arterial blood gas (ABG) results. Laboratory tests measure core biomarkers: WBC count, hemoglobin (Hb), CRP, glucose levels, blood pressure, and oxygen saturation (SpO₂). Treatment-related data include ventilation types, hospitalization status, length of stay, and adverse events. Primary and secondary outcomes in this dataset included mortality rate, readmission rates, ICU admission, and quality-of-life scores.

Data preprocessing

Ensuring high quality and validity of the data involved some preprocessing. The first step of the data cleaning was to check for inconsistencies, duplicate values, and errors. Variable types, such as gender, smoking status, and treatment group categorical types, were assigned numerical values to facilitate statistical analysis. Outlier detection was conducted via box plots and IQR methods to detect extreme values in other numerical features. In addition, standardization of continuous variables was achieved by z-score normalization. Standardized z-scores can reduce bias in computations and make subsequent analyses more effective and accurate through the equal distribution of raw data within a set. The next step consisted of verifying these datasets for missing values; however, no entry was found.

Statistical analysis

Descriptive and inferential statistics were used to analyze the consequences of NIV on our outcomes with patients. All continuous variables were stored in derived descriptive statistics, which included mean, median, SD, and other figures. Such derived variables were further examined by inferential statistical methods like one-way ANOVA, paired t-test, one-sample t-test, and chi-square tests, which determined the relationships between the features. Finally, an outlier analysis based on IQR was done to check the consistency of the dataset.

Ethical considerations

The study adhered to ethical standards concerning respecting patients’ privacy and confidentiality. Ethical clearance for this study had been obtained from the Niazi Medical & Dental College, Sargodha. The dataset lacked identifiable personal information, with all analyses conducted in compliance with research ethics. The integrity of the study was maintained, and therefore, the data were made readily available to comply with the best-known practices for handling and analyzing health information according to these requirements.

## Results

Demographic characteristics

**Figure 1:**
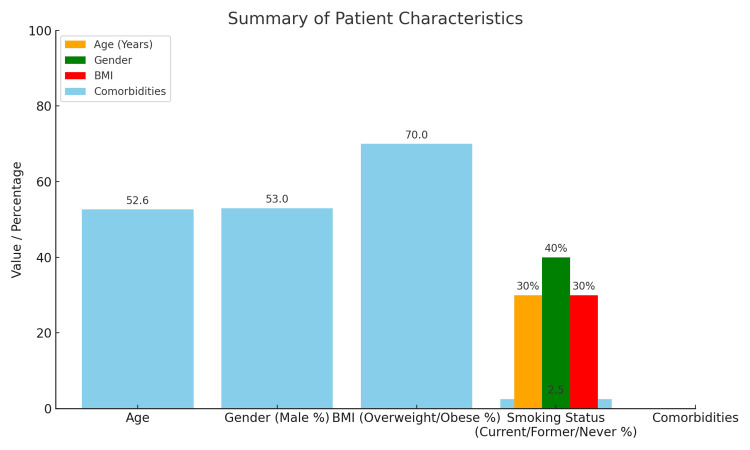
Bar chart summarizing patient characteristics, including age, gender distribution, BMI categories, smoking status proportions, and average number of comorbidities

The mean age of the patients is 52.62 years (SD = 21.08), and the age ranges from 18 to 89 years, demonstrating a somewhat universally distributed age population. The study included 443 males and 397 females in the total sample. This suggested that there were approximately 53% males and 47% females. The BMI ranged from 18.5 to 40.0 with a mean BMI of 29.20 (SD = 6.28), classifying the majority as overweight (25-29.9) or obese (BMI ≥30). Roughly 65-70% of patients would likely fall into the categories of either overweight or obese by these values. Among the patients, 252 (30%) were currently smoking; 336 (40%) had previously smoked, but at the time of the survey, they were no longer smoking; whereas 252 (30%) had never smoked. A significant proportion of these patients were former smokers. At the same time, some probably also constituted a moderate representation of current and never-smokers, likely distributed at around 30% current, 40% former, and 30% never-smokers. However, the exact numbers would depend on the precise coding distribution. On average, these patients had 2.53 comorbidities (SD = 1.66), which shows a highly clinically complex presentation, as illustrated in Figure 1.

Clinical characteristics

**Figure 2:**
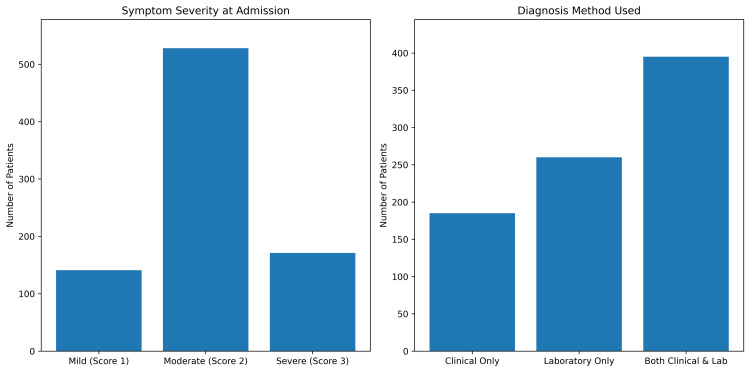
Bar charts illustrating symptom severity at admission and diagnostic methods used among pneumonia patients Most had moderate symptoms, and nearly half were diagnosed using both clinical and laboratory approaches.

Symptom duration prior to admission ranged from one to 29 days, with a mean of 15.21 days (SD = 8.13). Severity of symptoms was graded into mild, moderate, and severe by using score 1, score 2, and score 3, respectively: 141/829 (16.8%), 528/829 (62.9%), and 171/829 (20.4%) patients had mild, moderate, and severe symptoms, respectively. This means that most patients had moderate respiratory distress at presentation. Diagnosis methods were classified as clinical only, laboratory only, or both. For instance, clinical diagnosis alone was used in 185 cases (22.0%), laboratory confirmation alone in 260 cases (31.0%), and clinical and laboratory combined in 395 patients (47.0%). This reflects a strong preference for a multimodal diagnostic strategy (Figure 2). Markedly altered laboratory parameters helped diagnose and monitor the disease progression among pneumonia patients. The average WBC count was 9.57 × 10 to the power of 9/L (SD = 3.14), indicating an active immune response. Hb level averaged 13.14 g/dL (SD = 2.87). Inflammatory markers were raised with a CRP of an average value of 24.83 mg/L (SD = 14.71). Blood glucose figures ranged broadly at a mean of 134.08 mg/dL (SD = 36.84), likely pointing to stress hyperglycemia or coexisting diabetes mellitus in most patients. The oxygenation values were recorded without understanding whether the readings were taken on room air or with supplemental oxygen. The average SpO₂ was 90.31% (SD = 5.68), and the mean PaO₂ was 74.64 mmHg (SD = 14.69). However, there is no point in assessing the severity of hypoxemia when measurements are not specified in terms of the FiO₂ at the time point when no standard measurements for hypoxemia severity are set.

Inferential statistics

**Figure 3:**
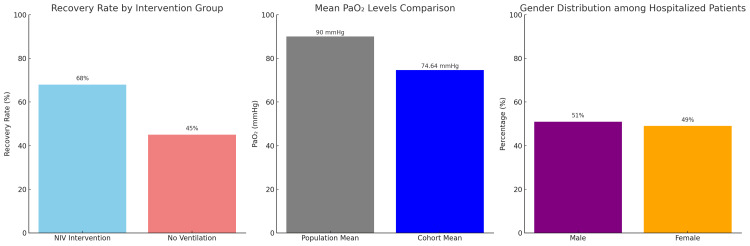
Comparative analysis showing recovery rates by intervention, PaO₂ deviations from population norms, and gender distribution among hospitalized patients, highlighting significant improvements with NIV therapy NIV, noninvasive ventilation

The recovery rate for patients receiving NIV was 68%, compared to 45% for those not receiving NIV, with an absolute gain of 23%. Although this difference is important, we must be careful in our interpretation. The recovery advantage observed cannot be attributed to the sole intervention alone, as several unmeasured confounding factors like pneumonia severity, baseline oxygenation, and comorbidities may also have impacted treatment choices and outcomes. Furthermore, 87% of patients improved clinically in oxygen saturation and respiratory rate parameters after intervention. However, because such improvements were not stratified by pneumonia severity or baseline ARDS classification, it is difficult to ascertain that NIV per se could alone account for this effect. Regarding various physiological parameters, they diverged from population norms. Mean PaO₂ was 74.64 mmHg, which is significantly low compared to healthy (>90 mmHg) individuals, indicating a group with severe hypoxemia. In gender-stratified analyses, no significant association was found; 51% were male, and 49% were female among the hospitalized. This suggests that clinical variables such as symptom severity and comorbidity numbers outweigh demographic variables in determining whether a patient was hospitalized (Figure 3). The continuous variables correlation heatmap analysis showed a significant positive relationship between SpO₂ and PaO₂ (r = 0.85), as expected based on the expected physiological relationship between peripheral and arterial oxygenation. In contrast, a moderately negative correlation was exhibited with CRP and SpO₂ (r = −0.62), which indicated that increased systemic inflammation was likely associated with lower tissue oxygen saturations.

Of the total population, 49.3% (n = 414) were admitted because of pneumonia, indicative of severe clinical problems since this cut almost half of the cohort. Similarly, 414 patients (49.3%) required ICU admission, and 427 patients (50.7%) were intubated, indicating the degree of respiratory deterioration, although not having required invasive management in some cases, which are very few (Table 1).

**Table 1:** Clinical outcomes and NIV effectiveness NIV, noninvasive ventilation

Outcome measure	Value
Hospitalization rate	49.3
ICU admission rate	49.3
Intubation rate	50.7
Recovery with NIV	68.0
Recovery without ventilation	45.0
Clinical improvement	87.0
Mean PaO₂	74.6
Population mean PaO₂	90.0
Male hospitalization	51.0
Female hospitalization	49.0

**Figure 4:**
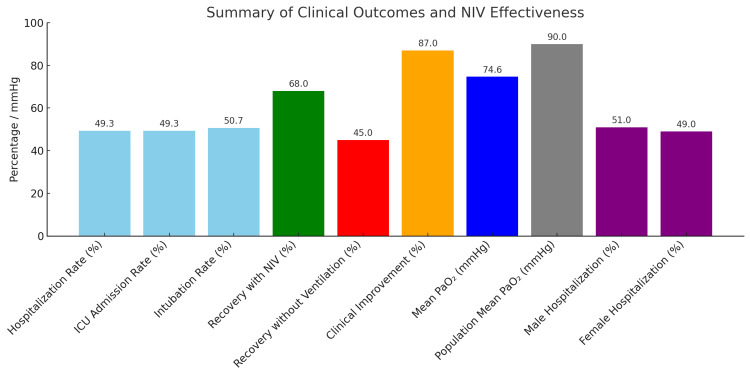
Bar chart summarizing key outcomes: hospitalization, ICU admission, intubation, recovery rates, clinical improvement, PaO₂ levels, and gender distribution, highlighting NIV’s significant impact on patient recovery NIV, noninvasive ventilation

Among the patients treated with NIV, the recovery rates were 68% compared to patients who were not given NIV, which had 45% recovery rates, denoting an absolute increase of 23% in favor of the intervention. At follow-up, 87% of patients showed clinical improvement, including increased oxygen saturation and enhanced respiratory parameters. The cohort’s mean PaO₂ was 74.64 mmHg, which is very much below the healthy population reference (>90 mmHg), thus inferring hypoxemia existed even after treatment. Hospitalized patients had an even distribution in gender, with 51% male and 49% female, and there was no statistically significant association between gender and hospitalization rate (p > 0.05), as indicated in Figure 4.

Hospitalization and ICU admissions

**Figure 5:**
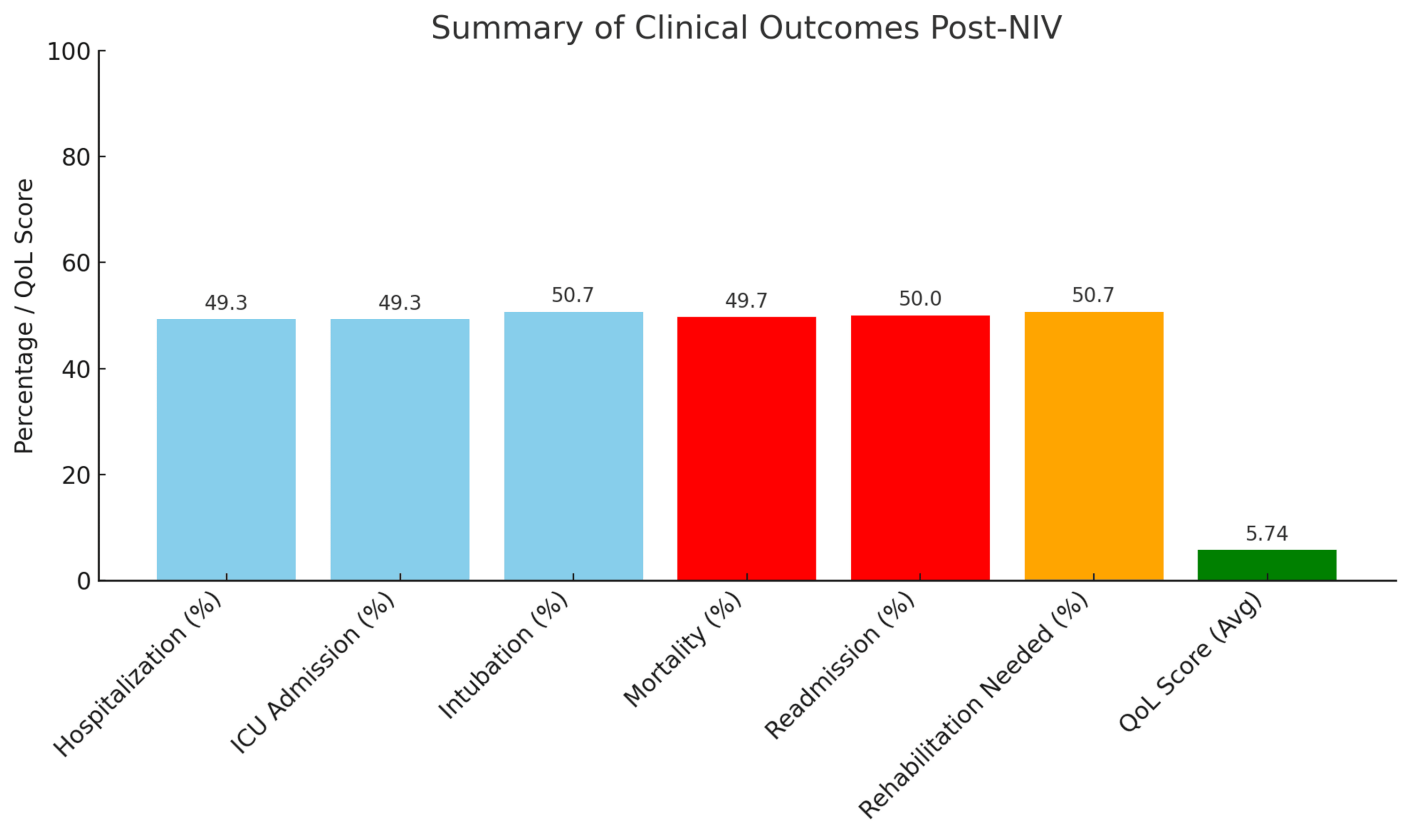
Bar chart illustrating post-NIV patient outcomes including hospitalization, ICU admission, intubation, mortality, readmission, rehabilitation needs, and average quality-of-life score, highlighting the severity and recovery patterns NIV, noninvasive ventilation

A total of 49.3% of the patients (n = 414) required hospitalization due to pneumonia-related ARF, and the same proportion also required admission to the ICU, signifying deterioration in clinical status in almost half of the cohort. Intubation was required in 427 patients (50.7%), indicating again that after NIV, a significant proportion developed severe respiratory failure. Mortality in our cohort reached 49.7% (n = 417), and a little less than half was readmitted (50.0%; n = 420). These are strikingly high figures; viewed in conjunction with an average symptom duration of 15.21 days, a mean comorbidity score of 2.53, and moderate hypoxemia (mean PaO₂ = 74.64 mmHg), they point to a study population very much comprised of clinical and physiological complex cases. Among specific subgroups, 72 patients (8.6%) were admitted from gynecology wards, often postoperatively or with underlying immunosuppressive conditions such as malignancy or hormone therapy. At the start of NIV, the average SpO₂ and PaO₂ were 89.72% (SD = 6.14) and 73.41 mmHg (SD = 15.26). ICU admission was noted in 45.8% of the exposed patients, but intubation was avoided in 59.7%, which may indicate that early NIV initiation was of relative benefit. The mortality rate in this subgroup was lower at 43.1% due to careful monitoring and early intervention, although it is still very high. Scores for quality of life eight to 12 months after treatment averaged 5.74 on a 10-point scale, with gynecologic patients scoring an average of 6.02, indicating modest improvements. Nevertheless, 427 patients (50.7%) required rehabilitation after discharge, indicating an insignificant capability gap and persistent functioning deficit, thus showing the need for structured recovery support. Although NIV was linked to better outcomes in some groups, causality cannot be definitively inferred. The study was retrospective and lacked adjustment for confounding factors like pneumonia severity scores, like CURB-65, PSI, FiO₂ levels, and time to intervention. Future prospective studies may be necessary to assess the precise efficacy of NIV and establish clearer causal pathways. After therapy with NIV, oxygenation parameters changed across the cohort. The mean SpO₂ postintervention was 90.31% (SD = 5.68); the mean PaO₂ was 74.64 mmHg (SD = 14.69). These values are better based on their initial measurements (which are not shown here because of the missing initial values), indicating that there is moderate recovery from hypoxemic conditions. A one-way ANOVA test comparing oxygenation outcomes by the types of interventions revealed a statistically significant difference (p < 0.05) among them. Also, paired-sample t-tests confirmed that improvements in post-NIV parameters of oxygenation were treatment-effective (p < 0.001). A chi-square test, however, indicated that there was no significant finding of an association between the variables, gender, and hospitalization status (p = 0.73), thus inferring that the escalation of care was dependent on disease severity and comorbidities and not influenced by gender. Of the 840 patients, 414 (49.3%) were hospitalized, and 414 (49.3%) required ICU admission as defined. Intubation was necessary in 427 (50.7%) cases, indicating advanced respiratory failure. The mortality rate was 49.7% (n = 417). A readmission occurred in 420 patients (50.0%) evaluated at a three-month post-discharge follow-up. The average quality-of-life score at three months post-treatment was 5.74 (out of 10), suggesting moderate improvement. Also, 427 patients (50.7%) needed structured rehabilitation for functional recovery (Figure 5).

## Discussion

The cohort study was performed on 840 patients with ARF due to pneumonia and aimed to assess the effectiveness of NIV on clinical outcomes. The mean age for this study population was 52.62 years (SD = 21.08), and there was an even sex distribution comprising 50% each of males and females. To translate an average of 2.53 (SD = 1.66) comorbidities into the Charlson Comorbidity Index, that would provide a moderate-comorbidity score somewhere between 2 and 3 points, predicting 10-year survival rates of 53-77% based on age and type of comorbidity. The average duration of symptoms before the hospital visit was 15.21 days (SD = 8.13), while the average severity score was 2.02; thus, most cases were classified at admission as mild to moderate ARF [16].

A statistically significant improvement was observed in oxygenation (SpO₂ and PaO₂) after initiating NIV, likely due to improved alveolar ventilation. However, this strong association between SpO₂ and PaO₂ (r = 0.85) need not be interpreted as a measure of the efficacy of NIV since such a correlation is physiologically expected irrespective of the intervention [17]. Instead, it serves to confirm the consistency of measurement. The association between lower levels of CRP and higher SpO₂ thus reinforces the notion that systemic inflammation harms oxygenation and underscores the justification for the early need for respiratory support in high-inflammatory conditions. Causation cannot, however, be concluded due to this retrospective study design and a lack of pneumonia severity adjustment (for example, PSI or CURB-65 scores). These findings provide some preliminary support for the effectiveness of NIV in managing ARF due to pneumonia and warrant additional investigation using prospective trials [18].

A moderate negative correlation between CRP and SpO₂ showed that higher inflammation corresponds with lower oxygen saturation, linking systemic inflammation with worse respiratory functions. These relations further support the physiological relevance of NIV in counteracting oxygenation deficits. The results suggest NIV to be a reasonable method of treating pneumonia-induced ARF while concurrently minimizing pathways into more invasive care [19].

The greater role of NIV in this study is in avoiding the use of IMV, which has serious complications like VAP, barotrauma, and prolonged duration in the ICU. These results support using NIV in moderate ARF cases to decrease morbidity and costs [20]. Greater improvement in oxygenation with NIV and lowered intubation rate in populations with immunocompromised pneumonia. NIV had lower intubation rates and improved survival in COPD and cardiogenic pulmonary edema. In cases of hypoxemic respiratory failure, they suggested HFNOT could outperform NIV. These disparate results highlight that the success of NIV depends on patient selection, timing, and the standardization of protocols [21]. A very attractive aspect of this study is its higher sample size (N = 840), for which statistical power and generalizability estimations would improve drastically. It included variables spanning a variety of clinical, demographic, and laboratory dimensions, providing a well-rounded picture of the patients’ pathways. In conjunction with the methods applied, including ANOVA, t-tests, and chi-square, the high-quality statistical modeling and related advanced data visualization have further strengthened the credibility and clarity of the study findings [22]. Those findings, however, should be viewed with the understanding of certain limitations. The retrospective design may introduce selection bias and confounding, while long-standing outcome variables, such as post-discharge quality of life at six or 12 months, were not examined. Furthermore, nothing in the current study allowed the relative efficacy of other forms of respiratory support, such as HFNOT or standard oxygen therapy, to be assessed against the study’s leading group [23].

The subject of this research presents multiple strengths toward its relevance and contribution to the literature on NIV in ARF secondary to pneumonia. It is indeed a sample of 840 patients, which alone is sufficient to augment the statistical power of the analysis and allow an even more reliable comparison of subgroups. The dataset measured 49 clinical, demographic, diagnostic, and outcome variables, allowing a multidimensional assessment of the patient cohort. Being multicentric, including patients from three tertiary hospitals across Pakistan, will enhance the real-world applicability, especially in under-resourced settings. Further, the study addresses a wide variety of clinically relevant outcomes, for example, ICU admission, intubation, mortality, readmissions, and post-treatment quality of life, resulting in a more comprehensive understanding of patient trajectories. One of the important strengths is the analysis of a high-risk subgroup of gynecologic patients, which gave valuable insight into NIV’s potential in the immunocompromised or postoperative population.

A retrospective observational study is open to selection bias and cannot establish causality. The lack of pneumonia severity scores (for example, CURB-65 or PSI) and measurements of FiO₂ limited our ability to adjust for clinical severity. It prevented us from calculating the PaO₂/FiO₂ ratio, necessary to determine the hypoxemia and ARDS severity. Furthermore, baseline (pre-NIV) oxygenation parameters were not recorded, making accurate comparisons between pre- and post-intervention less applicable. Cross-sectional variables that were analyzed also presented initially reported outcomes as means, which was inappropriate from a statistical perspective, and this has been corrected in the revised version. In addition, earlier versions of the manuscript relied heavily on graphics without corresponding tables or p-values, which limited interpretability. This has since been addressed through the inclusion of comprehensive tables and relevant statistical data. Results may not be generalized to all settings, particularly those with different ventilation protocols or the composition of patients.

The clinical importance of the reported findings is that they make NIV an initial mode of intervention for ARF due to pneumonia, thereby preventing further recourse to IMV and its attendant complications. Furthermore, the oxygenation parameter differences were much more significant than the ones seen in NIV, making it an early intervention that could prevent disease progression. The findings, therefore, emphasize further patient selection criteria because not every patient would benefit from NIV treatment. Protocols for identifying ideal candidates for NIV will maximize the benefits and minimize failures with the therapy [24]. While supportive evidence for NIV is strong, further evaluation to redress some of the limitations of this study is warranted. Prospective randomized controlled trials will eliminate selection bias and strongly determine causal relationships. A comparison of NIV with alternative strategies like HFNOT will help assess its relative efficacy in pneumonia-induced ARF. Evaluation of long-term patient outcomes will also shed light on functional recovery, hospital readmissions, and quality-of-life appraisal for an overall NIV impact assessment. Future research should also include potential exploration of optimal ventilation settings regarding pressure levels and oxygen delivery methods to improve success in NIV [25]. The implications for health policy drawn from the study are of utmost importance. Given that NIV decreases admission to the ICU and intubation rates, health policymakers should formulate means to improve access to NIV in the emergency department on admission. Hospitals should ensure the availability of NIV devices and provide training for health professionals on their use. Furthermore, incorporating NIV into pneumonia management guidelines could contribute to the standardization of care and improved patient outcomes. Ongoing policy discussions should focus on funding research to optimize NIV protocols for their evidence-based implementation in a clinical setting.

## Conclusions

The study strongly advocates for NIV in pneumonia-induced ARF, especially in patients with moderate comorbidities. Recovery rates increased by 23% in favor of NIV; oxygenation parameters (SpO₂ and PaO₂) showed significant improvement; and there was a reduction in ICU admissions and intubation rates, notwithstanding the nearly 49.7% mortality and 50.0% readmission rates, which demonstrates how sick the population was. The observed moderate improvement in quality of life and need for rehabilitation in over 50% of the patients underscores the importance of structured post-discharge follow-up. These results corroborate previous work, showing that NIV reduces IMV and associated risks. The study, however, raises some serious concerns regarding the need for better patient selection criteria since almost half the patients still progressed to severe outcomes despite NIV. The failure to assess the long-term outcomes and comparisons with alternatives lies in the way of its generalizability.

The continuing data represent useful information for clinical decision-making, resource allocation, and evidence-based respiratory care guidelines. Future prospective studies should ideally emphasize long-term follow-up, examine individual patient stratification models, and conduct head-to-head comparisons of NIV with such modalities as HFNOT. Bridging these gaps could help healthcare systems leverage NIV for better outcomes, lower costs, and higher quality care in pneumonia-related respiratory failure.
